# From “doing alone” to “working together”—Research on the influence of spiritual leadership on employee morale

**DOI:** 10.3389/fpsyg.2023.992910

**Published:** 2023-03-15

**Authors:** Juan Jiang, Zhixiao Ye, Jing Liu, Wasi Ul Hassan Shah, Zahid Shafait

**Affiliations:** ^1^Department of Financial Management, Business School, Ludong University, Yantai, Shandong, China; ^2^Property Management Department, School of Management, Zhejiang Shuren University, Hangzhou, Zhejiang, China; ^3^International Economy and Trade Department, Business School, Ludong University, Yantai, Shandong, China; ^4^School of Management, Zhejiang Shuren University, Hangzhou, Zhejiang, China; ^5^College of Teacher Education, Zhejiang Normal University, Jinhua, Hangzhou, China

**Keywords:** self-efficacy, interpersonal trust, sustainable development, spiritual leadership, employee morale

## Abstract

Spiritual leadership is a beneficial attempt to implement organizational strategy and sustainable development by improving employees’ personal spiritual needs, stimulating employees’ intrinsic motivation, sense of responsibility, and calling in the work process. We theoretically illustrate that spiritual leadership has a significant positive impact on employee morale. The factors of personal self-efficacy and interpersonal trust play an intermediary chain role in this process. SPSS 24.0 and Process3.5 were used to test the mediating effect using multilevel regression analysis and the bootstrap method. Survey data collected from 278 employees from Chinese organizations supported our proposed hypotheses. The research verifies it is necessary to pay attention to improving the spiritual needs of leaders and employees in organizational development. Through the cultivation, spiritual leadership not only can stimulate the cohesion of the organization and the intrinsic motivation of the employees, but also is of great significance to enrich the spiritual life of the organization members.

## 1. Introduction

In the busy modern life, work is no longer just a means of making living. People invest time and energy in their work and it has become an important part of the connection between people. Work has also become the most important way to meet the material needs and spiritual needs for modern people. As to many people, the time spent on families is decreasing, while that spent on work is increasing. Terms such as “Neijuan^1^” and “996^2^” have emerged. In the process of work, people release their potential, complete their work goals and obtain material rewards and spiritual satisfaction to realize their self-worth. With the enrichment and satisfaction of material life, people urgently need to obtain spiritual recognition and satisfaction. Spiritual research in the workplace has emerged and flourished, with increasingly abundant research results (Benefiel et al., 2014).

In spite of rich theoretical contents, modern organization motivation is difficult to play its role in the practice process, and organizations face “motivation strange phenomenon “—managers clearly attach great importance to motivation problems also invested a lot of resources and energy, but gained no essential performance promotion from employees, and even their resistance and boredom to the management. The idea of “eating from a big pot” emerged. Many employees are satisfied with the existing state of affairs and reluctant to move forward in organizations (Zhu, 2014). Although stimulating employee morale has always been a problem in organizations, it is particularly urgent and important today. According to a report published on April 2020 by the Association of Human Resource Management from America., 2/3 of employers agree maintaining employee morale has always been a huge challenge; 35% of employers face employee productivity changes, while companies with more than 500 employees witness more challenging than small or medium-sized employers. 73% of residential/food service/healthcare facility staff had low morale (Pattnaik and Jena, 2021).

Leaders must recognize that employees have spiritual needs, as well as physical, mental, and emotional needs (Latham, 2013). At the same time, during the organizational reform, the distrust generated by drastic changes lead to low morale and spiritual loss (Kinjerski and Skrypnek, 2004). In particular, the COVID-19 pandemic has greatly changed people’s work styles and lifestyles. In the face of drastic changes, people’s attitudes and emotions toward work become more complex: on one hand, they have to deal with the huge work pressure generated in the process of social and organizational changes; on the other hand, people urgently need to realize their self-worth through work achievements.

In 2021, LinkedIn conducted a survey of the most concerned issues of job seekers around the world, and found that the first value proposition of employees is a good balance between work and life, followed by excellent salary and benefits. People no longer regard work as a means to make a living, but to gain respect and need, so as to obtain a higher level of life experience, which is also a beneficial discussion of the satisfaction and improvement of human spiritual needs^3^. In this context, the issues of motivation and trust become major issues for the survival and development of organizations, especially for enterprises, and these issues can be offset by the positive effects of spiritual in the workplace (Kinjerski and Skrypnek, 2004; Karakas and Sarigollu, 2019; Ali et al., 2020, 2022a,b).

Although spiritual leadership theory is put forward in Western culture, it is also suitable in the context of Chinese culture (Chen et al., 2012; Chen and Li, 2013; Yang et al., 2014). Confucius said, “With good manners, people do not dare to disrespect; Good justice, the people dare not refuse; Good faith, then people dare not use the feeling “. It is the exact impact interpretation of spiritual leadership on the subordinates. Therefore, this paper tries to explain that spiritual leadership is directly and indirectly related to employee morale.

First, based on the self-determination theory(SDT), employee morale and performance are more influenced by intrinsic motivation(Deci and Ryan, 2000; Ryan and Deci, 2000). Experiencing spiritual leadership, employees feel caring and love from leaders, which is more likely to stimulate their intrinsic motivations. This stimulation can be explained by the positive effect of spiritual leadership on employee’s self-efficacy at the individual level and leads to promotion of employee morale.

Second, as the research combines workplace spirituality theory and leadership theory, spiritual leadership provides ideas and methods for leaders in organization to enhance spiritual needs. While they believe in the ability, integrity and kindness of their leaders, followers are easier to trust and are willing to engage in risky behavior (Mayer et al., 1995). Also, Avolio et al. (2004) believe that the trust from leaders is related to the positive attitudes of followers. Therefore, spiritual leadership increases employee morale by improving interpersonal trust within organizations.

Additionally, spiritual leaders enrich the spiritual needs and the members’ spiritual world by using vision, hope and altruistic love to ascend their intrinsic motivation to form the sense of calling and responsibility in the workplace so as to achieve a higher level of life experience. The crossover principle of conservation of resources (COR) is invoked to explain the positive relationship between spiritual leadership and employee morale. We conceptualized spiritual leadership as contextual resources while the self-efficacy and interpersonal trust as the employees’ personal resources. Spiritual leaders’ core values and vision associated with this type of leadership can transmit to employees (Ali et al., 2022a).

We developed a theoretical model to illustrate how spiritual leadership influence on employee morale stressing on the influence mechanism and process. Spiritual leadership positively affects employee self-efficacy, further positively affects interpersonal trust within the organization, and ultimately improves employee morale. At the same time, we demonstrated all the hypothesis empirically using the data from the questionnaire survey from the organization members of China.

## 2. Theoretical basis and research hypothesis

### 2.1. Spiritual leadership

Spiritual leadership, as a more spiritual and faith-based leadership type, focuses on the organization’s vision. The development of workplace spirituality, character ethics, positive psychology, and other theories provides a consensus on the values, attitudes, and behaviors necessary for positive human health and well-being, thus enriching the theoretical content of spiritual leadership.

As for spiritual leadership, scholars have made different definitions from different perspectives (Wu and Xiang, 2021), including the perspective of behavioral purpose of leaders (Aslan and Korkut, 2015), organizational perspective (Thompson, 2004), individual perspective (Reave, 2005), and dimensional content (Sendjaya, 2007). The widely accepted definition originates Fry’s behavioral process perspective, which defines spiritual leadership as the causal leadership theory that spiritual leadership essentially achieves organizational change through intrinsic motivation and learning (Fry, 2003; Fry et al., 2005, 2017). Spiritual leadership involves intrinsically inspiring values, attitudes, and behaviors in oneself and others to obtain a spiritual sense of existence through a sense of calling and belonging (Fry et al., 2005). Spiritual leadership sources from inner life; altruistic love is the culture foundation while hope/belief is the impetus for the vision of serving key stakeholders (Fry et al., 2017; Yang and Fry, 2018; Ali et al., 2020).

To sum up, the spiritual leadership has three key features: (1). A high level of moral values, such as integrity, honesty, caring, notarization, etc.; (2). Encouraging and guiding others, valuing mutual connections with subordinates and collaborators; (3). Motivating persons to achieve organizational vision and mission, to explore the significance of the work (Wu and Xiang, 2021).

Many scholars adopted empirical methods to analyze and study spiritual leadership’s antecedents and influence factors and drew abundant conclusions (see Table 1 for details).

**Table 1:** Partial results of empirical studies on spiritual leadership.

Perspective	Antecedents	Influence factors	Intermediary effect	Moderating effect
Individual	Characteristics altruism and trust Confucian values…	Employee behavior Job performance Psychology/attitude Career development…	Psychological capital Employees’ self-esteem Self-efficacy Employee cognitive Trust in leaders Professional ethics…	Gender Length of service Culture Perceived organization support Emotional intelligence…
Team	Common vision Incentive mechanism…	Team performance Team innovation…		
Organization		Organization performance Organization culture Organization transformation…		

The authors arranged referring to Wu Xiangfan, Xiang Yi. Review and Prospect of spiritual leadership in organizations. *Journal of Xinjiang University of Finance and economics*, 2021 (2): 13.

The research done by Chinese scholars on the variables of spiritual leadership mainly focuses on the individual level, including employees’ psychological capital, emotional commitment, proactive behavior, autonomous motivation, task performance, creativity and innovation, employees’ expostulation, retention will, and craftsman spirit. Scholars affirm the positive influence of spiritual leadership on employees. As for the mediating variables of spiritual leadership’s influence, the main conclusions of current research are: individual-level factors include psychological capital (Deng, 2016), autonomous motivation (Shi et al., 2018), psychological toughness (Wang and Wang, 2019), and psychological need satisfaction (Deng et al., 2021; Wu, 2021). Organizational-level factors include leader-member exchange (Meng et al., 2018; Ni et al., 2019), workplace spirituality (Deng et al., 2020; Ke and Ding, 2020), organizational identification etc.

Scholars believed that spiritual leadership has a positive impact on individual employees (Usman et al., 2021). While some scholars have proved that spiritual leadership has a negative impact on individual passive factors, as work alienation (Khan et al., 2019; Wang and Wang, 2019; Ali et al., 2022b).

But whether the positive impact on individuals can rise to the level of the organization, that is, the content and mode of the impact of spiritual leadership on the organization are not clear. At the same time, the contents of outcome variables, mediating variables, and moderating variables of various studies are overlapping and repeated, so it is urgent to clarify the logical mechanism of applying spiritual leadership to affect employees in organizations in the context of Chinese culture.

### 2.2. Employee morale

The theoretical content of employee morale definition includes needs psychology, needs hierarchy, “field” theory and organizational identity, etc. (Baehr and Renck, 1958; Huang et al., 2015). Although scholars have different perspectives and definitions of morale, they can reach a consensus on the essence of morale. That is an attitude, a kind of enthusiasm for labor, manifested as a state of mind. Employee morale at work results from the interaction between individuals and the group. In this process, individual needs are met, and individuals identify with the organizational goals, resulting in a harmonious and consistent situation. Therefore, individuals are willing to cooperate with other members of the group (Huang et al., 2015).

Employee morale includes a mix of emotions and attitudes that can generate high levels of energy, spirit, and willingness to work to improve organizational performance (Pattnaik and Jena, 2021). The positive feelings of employees, such as enthusiasm and willingness to cooperate clearly means high morale. Similarly, low morale depicts employees’ negative emotions, such as dissatisfaction, discouragement, and dislike of work, which leading to increased employee turnover (Kanimozhi and Vinothkumar, 2018). Compared with employees with low morale, employees with high morale are more willing to work harder and are more committed to organizational goals (Bowles and Cooper, 2009).

By establishing the consistency between employees’ personal and organizational values, spiritual leadership enables employees to recognize and respect spiritually. The employees are more willing to regard work as a requirement of self-realization rather than a simple means of making a living, and they are willing to devote more time and energy to work. Spiritual leadership can keep leaders focusing and motivating employees effectively, thus solving the low morale of employees (Hyson, 2013). Spiritual leadership is the “beacon” of employee career development, which guides and promotes employee career development (Yang et al., 2014). As spiritual leadership attaches importance to employees’ satisfaction at the spiritual level, it significantly impacts employees’ happiness, satisfaction, organizational commitment, and other positive emotions. Spiritual leadership has a significant positive impact on career calling (Shi et al., 2018), positively affecting employees’ work experience. Therefore, spiritual leadership can improve the employee morale, and then we put forward hypothesis 1: spiritual leadership has a significant positive impact on organizational staff morale.

### 2.3. Interpersonal trust

Trust is defined differently in different areas (Rousseau et al., 1998): Economists tend to see trust calculable (Williamson, 1993), or institutionalized (Hicks et al., 1974); Psychologists generally build their own evaluation of trust based on the attributes of the trustor and trustee and value a series of internal perceptions generated by personal attributes (Rotter, 1968; Tyler and Lind, 1990). As the complex meaning of trust, this paper only discusses the content of interpersonal trust within the organization, including the trust of employees in leaders and the trust between colleagues.

Leaders in organization plays an important role in the process of trust-building. Trust in leaders and professional ethics play a mediating role in the relationship between spiritual leadership and organizational virtues (Taboli and Abdollahzadeh, 2016). In Chinese enterprises, some characteristics of leaders (such as moral quality, paternalism, etc.) have a profound impact on the psychology and behavior of employees (Zhu and Akhtar, 2014). Spiritual leadership can inspire employees from the spiritual level, including vision, hope, and generous love, to form an atmosphere of unity and enhance employees’ confidence in themselves, leaders, colleagues and the organization. Spiritual leadership can influence employees’ expostulation through the chain mediating effect of organizational atmosphere and psychological security (Wang et al., 2017), which shows that spiritual leadership positively impacts the spiritual atmosphere within an organization. Therefore, hypothesis 2 is proposed: spiritual leadership has a significant positive impact on interpersonal trust.

Trust in leaders is associated with positive attitudes of followers, which in turn is associated with positive behaviors (Avolio et al., 2004). It is the most direct, economical and efficient way for leaders to improve the effectiveness of the organization (Cao et al., 2008). Positive interpersonal trust will form an upward working atmosphere. Employees, perceiving this atmosphere, will have the requirements of self-value realization and work hard, thus improving organizational staff morale as a result. Therefore, this paper proposes hypothesis 3: interpersonal trust has a significant positive impact on employee morale.

### 2.4. Self-efficacy

Self-efficacy is defined as confidence in their ability to mobilize the motivation, cognitive resources, and course of action to perform a particular action in a given environment (Bandura, 1977, 2002). As a subjective feeling of evaluating one’s own ability, self-efficacy will affect employees’ attitude, emotions, and behavior performance at work (Chen and Li, 2017). Employees with high self-efficacy show more positive job performance (Stajkovic and Luthans, 2010). Higher self-efficacy among employees in an organization will lead to overall positive motivation and pursuit and higher employee morale. Therefore, hypothesis 4 is proposed: self-efficacy has a significant positive impact on employee morale.

Employees with high self-efficacy will show strong interpersonal coordination and communication skills and be more active and proactive in their communication with leaders and colleagues (Yan and Chen, 2009). Such positive communication will enhance the understanding among members, thus providing a favorable premise for forming a positive trust relationship. Positive communication will also be helpful to build a good working atmosphere, conducive to forming a trust. Therefore, hypothesis 5 is proposed: self-efficacy has a significant positive impact on interpersonal trust.

Since spiritual leadership focuses on stimulating employees’ spiritual needs to improve their intrinsic motivation and value, as well as their work experience and status, it is certainly conducive to developed employees’ positive psychological state and improve their psychological capital level (Yang et al., 2015). Therefore, hypothesis 6 is proposed: spiritual leadership significantly positively impacts self-efficacy.

Based on theoretical and empirical analysis, researchers believe that transformational leadership positively impacts team effectiveness through the cognitive trust of leaders, while service leadership positively impacts team psychological security through emotional trust; they both improve team performance, and trust’s mediating effect is proved (Schaubroeck et al., 2011). According to the research content of hypotheses 1 (Spiritual Leadership ⇒ Employee Moral), 2 (Spiritual Leadership ⇒ Interpersonal Trust), and 3 (Interpersonal Trust ⇒ Employee Moral), hypothesis 7 is proposed: interpersonal trust plays a mediating role between spiritual leadership and employee morale.

Spiritual leadership stimulates positive behaviors and attitudes of followers by enhancing their psychological capital level (Yang et al., 2015). A strong sense of self-efficacy can produce high motivation and excellent performance when employees’ personal and organizational goals are consistent (Appelbaum and Hare, 1996). Spiritual leadership meets the spiritual needs of employees and confirms that employees’ goals are consistent with the organization’s vision. This can enhance employees’ morale and improve the organization’s performance. Therefore, based on hypotheses 1 (Spiritual Leadership ⇒ Employee Moral), 4 (Self-efficacy ⇒ Employee Moral), and 6 (Spiritual Leadership ⇒ Self-efficacy), hypothesis 8 is proposed: self-efficacy plays a mediating role in the relationship between spiritual leadership and employee morale.

Therefore, based on hypotheses 5 (Self-efficacy ⇒ Interpersonal Trust), 7 (Spiritual Leadership ⇒ Interpersonal Trust ⇒ Employee Moral), and 8(Spiritual Leadership ⇒ Self-efficacy ⇒ Employee Moral), hypothesis 9 is proposed: Self-efficacy and interpersonal trust play an intermediary chain role between spiritual leadership and employee morale.

**Figure 1:**
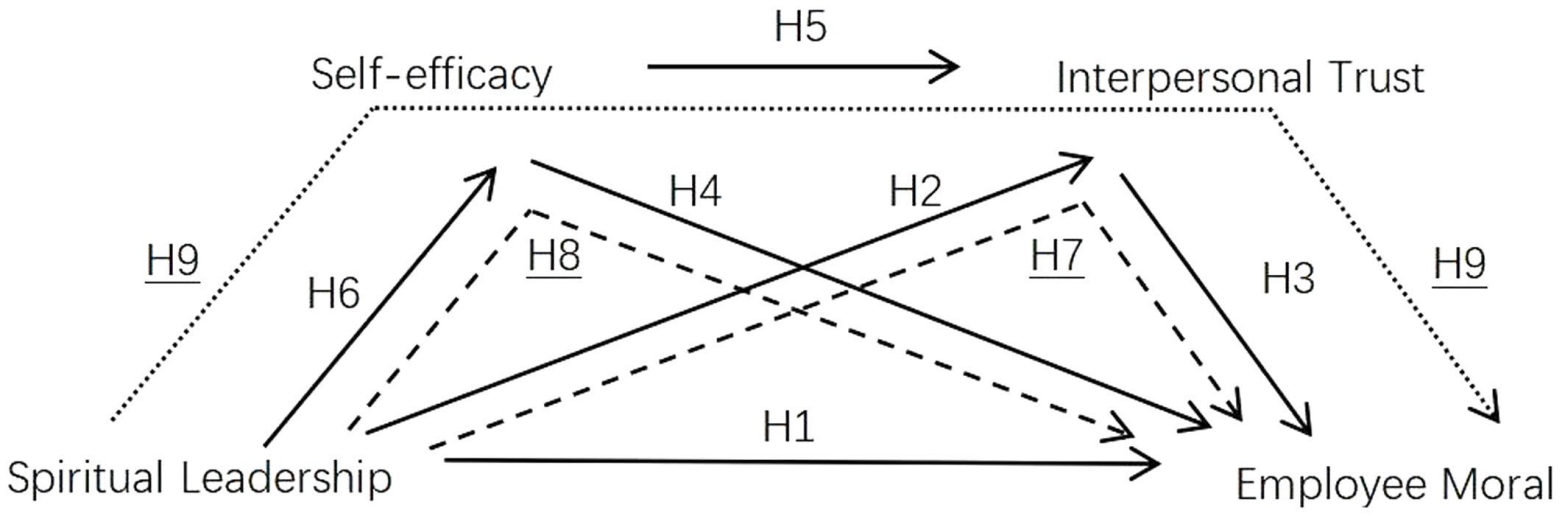
The research hypothesis model diagram: the relationship between spiritual leadership and employee morale: a chain mediation of self-efficacy and interpersonal trust.

Based on the above analysis, We draw the model diagram of the research hypothesis in Figure 1.

## 3. Research design and analysis

### 3.1. Data collection

This study adopts the method of a questionnaire survey to collect a wide range of samples from specific people in specific industries, which is relatively low cost (Heeringa et al., 2017; Rasool and Samma, 2020; Shafait et al., 2021). We invited the university alumni to help us complete the questionnaire. Because alumni involve in different majors and industries, it can be considered random questionnaires. We asked two college English teachers to help us translate all the items of the questionnaire together and performed preliminary testing. The written expression of some items was adjusted based on communication with respondents in the preliminary test. All questionnaires were conducted through the Questionnaire Star platform^4^ and post-interview between June and August, 2020.

The spiritual leadership questionnaire is developed by Fry et al. (2017), including 17 items from three dimensions: vision, hope, and altruistic love. The employee morale questionnaire is developed by Griffith’s research (Griffith, 2010), and we select the three dimensions with the highest proportion of principal component analysis among the six dimensions, including supervision, job satisfaction, and job involvement, with 26 items. The self-efficacy questionnaire is developed from the self-efficacy dimension of Psychological Capital by Luthans et al. (2007) and contains seven items in a single dimension. All the three scales were Likert 7-point scales in which 1 = “strongly disagree” and 7 = “strongly agree.” As the questionnaire on interpersonal trust, 18 items are selected for the two dimensions including trust in leaders from Nyhan and Marlowe (1997) and trust in colleagues developed by Seers et al. (1995). This questionnaire asked the sample to choose the number from the scale in which 1 = “nearly zero “and 7 = “nearly 100%”.

Respondents are required to provide information of gender, length of the time employed, working positions, their organization’s nature and industry. A total of 278 valid questionnaires were collected from June to August 2020, including 105 males (37.8%) and 173 females (62.2%). 73 (26.3%) have worked for 1–5 years, 89 (32.0%) have worked for 6–10 years, 67 (24.1%) have worked for 11–15 years, 21 (7.6%) have worked for 16–20 years, and 28 (10.1%) have worked for more than 20 years. There are 134 ordinary employees (48.2%), 67 grass-roots managers (24.1%), 57 middle managers (20.5%) and 20 senior managers (7.2%). There are 100 employees in state-owned enterprises (36.0%), 108 employees in private enterprises (38.8%), 17 employees in wholly foreign-owned enterprises (6.1%), and one employee in a sino-foreign joint venture (0.4%), and 52 employees in non-profit organizations (18.7%).We classified the organizational industries according to the Classification of Industries in the National Economy, which began on March 29,2019 in China. The sample involved all 16 industries. Among them, the most involved is in education, finance, public services, and manufacturing. The description of the questionnaire sample characteristics was shown in Table 2.

**Table 2:** Description of the questionnaire sample characteristics.

	NO.	Items	Frequency	Percentage(%)
Gender	1	Male	105	37.8
	2	Female	173	62.2
Total				100
Length of the time employed	1	1–5 years	73	16.3
	2	6–10 years	89	32.0
	3	11–15 years	67	24.1
	4	16–20 years	21	7.6
	5	More than 20 years	28	10.1
Total				100
Position	1	Ordinary employees	134	48.2
	2	Grass-roots managers	67	24.1
	3	Middle managers	57	20.5
	4	Senior managers	20	7.2
Total				100
Organizational nature	1	State-owned enterprises	100	36.0
	2	Private enterprises	108	38.8
	3	Wholly foreign-owned enterprises	17	6.1
	4	Sino-foreign joint venture	1	0.4
	5	Non-profit organizations	52	18.7
Total				100
Industry	1	agriculture, forestry, animal husbandry, and fishery	3	1.1
	2	mining	3	1.1
	3	manufacturing	33	11.9
	4	construction	10	3.6
	5	wholesale and retail	15	5.4
	6	transportation, storage, and postal services	11	4.0
	7	finance	52	18.7
	8	realty	8	2.9
	9	education	57	20.5
	10	accommodation and Catering	5	1.8
	11	resident services, repair, and other services	5	1.8
	12	scientific research and technology services industry	7	2.5
	13	information transmission, software, and information technology services	19	6.8
	14	culture, sports, and entertainment	1	0.4
	15	leasing and business services	7	2.5
	16	public services and management	42	15.1
Total				100

As multiple research variables are high-order variables and contain multiple dimensions, principal component variables of each dimension are extracted as dimension variables. Then the mean value of each dimension of the questionnaire is taken as the research variable. Some items with standard loading coefficients less than 0.6 (item 5 of the generous love dimension of spiritual leadership and item 1 of the morale supervision dimension) were removed in extracting principal components. All dimension items passed Bartlett’s sphericity test and were suitable for factor analysis. The specific data can be found in Table 3.

**Table 3:** Extraction of model variables and dimension factors, reliability and validity test table.

Variable name	Dimension name	KMO (*p* < 0.001)	Extraction sums of squared loadings % of variance	Standardized Clonbach *α* coefficient	CR	AV
Spiritual leadership	Vision	0.901	83.0%	0.976	0.949	0.787
	Hope	0.893	84.3%		0.954	0.807
	Altruistic love	0.904	84.0%		0.962	0.809
Employee moral	Supervision	0.962	76.7%	0.976	0.976	0.745
	Job involvement	0.879	77.9%		0.942	0.730
	Job satisfaction	0.869	79.6%		0.934	0.739
Interpersonal trust	Trust in leaders	0.934	88.1%	0.972	0.981	0.864
	Trust in colleagues	0.935	74.8%		0.962	0.719
Self-efficacy		0.906	72.8%	0.934	0.933	0.666

### 3.2. Reliability and validity tests

The reliability test of the questionnaire data shows that the α coefficients of spiritual leadership, employee morale, interpersonal trust, and self-efficacy are above 0.9, indicating the high-reliability quality of the data. Confirmatory factor analysis was conducted for each variable and dimension, and the corresponding combined reliability (CR value) and mean–variance extraction (AVE value) were both greater than 0.7 and 0.5, indicating good aggregation validity of data (see Table 3 for specific data).

### 3.3. Hypothesis test

In this research, spiritual leadership is taken as an independent variable, employee morale as a dependent variable, and self-efficacy and interpersonal trust as intermediary variables. The SPSS 24.0 software is employed to test the theoretical hypothesis through multiple rounds of hierarchical regression. And plug-in Process 3.5 is adopted to test Model 7, 8, 9, the integration of the chain intermediary, and the bootstrap method is used to repeatedly sample 5,000 times to calculate the 95% confidence interval, respectively. The results are shown in Tables 4, 5. In the regression process, the multicollinearity is tested, and the VIF coefficients are less than 2, indicating no multicollinearity between variables.

**Table 4:** Test results of intermediary effect model between spiritual leadership and employee morale.

	Self-efficacy			Interpersonal trust			Employee morale			Employee morale		
	*β*.	SE	*t*.	*β*.	SE	*t*.	*β*.	SE	*t*.	*β*.	SE	*t*.
Constant	–	0.214	0.462	–	0.142	1.548	–	0.137	0.63	–	0.082	−0.653
Gender	−0.117	0.097	−2.477^*^	−0.033	0.065	−0.951	−0.07	0.062	−2.073^*^	0	0.038	0.004
Working years	0.076	0.038	1.609	−0.052	0.026	−1.512	0.018	0.025	0.545	0.006	0.015	0.315
Working position	0.094	0.049	1.949	−0.02	0.033	−0.584	0.058	0.031	1.686	0.023	0.019	1.123
nature of organizations	−0.024	0.033	−0.504	0.007	0.022	0.215	−0.006	0.021	−0.18	0.002	0.012	0.088
Spiritual leadership	0.625	0.049	13.61 ^***^	0.459	0.042	10.657 ^***^	0.834	0.031	25.488 ^***^	0.331	0.029	10.999^***^
Self-efficacy				0.47	0.04	10.71 ^***^				0.255	0.028	8.289 ^***^
Interpersonal trust										0.456	0.035	12.783^***^
*R* ^2^	0.433				0.698		0.707			0.898		
*F* value.	F(5,272) = 41.509^***^			F(6,271) = 107.687^***^			F(5,272) = 134.379^***^			F(7,270) = 340.603^***^		

(1) *N* = 278; (2) ^*^p < 0.05, ^**^p < 0.01, ^***^p < 0.001.

**Table 5:** Analysis of intermediary effect between spiritual leadership and employee morale.

Terms	Effect	Boot SE	BootLLCI	BootULCI	Standardized effect quantity
H7:Spiritual Leadership⇒Self-efficacy⇒Employee Moral	0.152	0.014	0.121	0.174	0.159
H8:Spiritual Leadership⇒Interpersonal Trust⇒Employee Moral	0.200	0.032	0.141	0.263	0.309
H9:Spiritual Leadership⇒Self-efficacy⇒Interpersonal Trust⇒Employee Moral	0.128	0.02	0.088	0.166	0.134
H1:Spiritual Leadership⇒Employee Moral	0.797	0.031	0.736	0.858	0.503

Boot LLCI refers to the lower limit of 95% interval of bootstrap sampling, BootULCI refers to the upper limit of 95% interval of bootstrap sampling.

It can be seen from Table 4. that spiritual leadership has a significant positive impact on employee morale (Hypothesis 1 proved, *t* = 25.488). Spiritual leadership significantly positively impacts self-efficacy (Hypothesis 6 proved, *t* = 13.61). Spiritual leadership and self-efficacy significantly positively impact interpersonal trust (Hypotheses 2 and 5 proved, *t* = 10.657 and *t* = 10.71). Spiritual leadership, self-efficacy, and interpersonal trust significantly affect employee morale (Hypothesis 3 and 4 proved, *t* = 10.999/8.289/12.783). After adding the variables of self-efficacy and interpersonal trust, the impact of spiritual leadership on employee morale is still significant, and the explanatory ability of the equation to the dependent variable is enhanced (*R*^2^ increased).

According to the analysis results in Table 5, the confidence interval corresponding to each indirect path does not contain 0, indicating that the mediation effect is significant and the chain mediation is established. That is, self-efficacy plays a partial intermediary role between spiritual leadership and employee morale (Hypothesis 7 proved), interpersonal trust plays a partial intermediary role between spiritual leadership and employee morale (Hypothesis 8 proved), and self-efficacy and interpersonal trust play an intermediary chain role between spiritual leadership and employee morale (Hypothesis 9 proved).

## 4. Conclusion and recommendations

### 4.1. Conclusion

We theoretically analyze the positive correlation between spiritual leadership, employee morale, interpersonal trust and self-efficacy and established a theoretical model of the influence of spiritual leadership on employee morale, expressed spiritual leadership has a positive impact on employees’ self-efficacy from a personal perspective, then affects interpersonal trust, and ultimately affects employee morale. We also empirically prove the following conclusions based on questionnaire data from different industries in China: spiritual leadership has a positive impact on self-efficacy, interpersonal trust, and employee morale; self-efficacy and interpersonal trust play a separate intermediary role and an intermediary chain role between spiritual leadership and employee morale. The research proves the influence mechanism of spiritual leadership on employee morale: spiritual leadership has a positive impact on self-efficacy, which shows that spiritual leadership can improve employees’ spiritual needs, stimulate their intrinsic motivation and generate more positive work incentive from the individual perspective; spiritual leadership and self-efficacy positively affect interpersonal trust, which shows that spiritual leadership can produce a more trusted working atmosphere within the organization; spiritual leadership, self-efficacy and interpersonal trust have a positive impact on employee morale, which reveals that spiritual leadership can stimulate the cohesion and sense of calling at the organizational level from the individual and collective levels, enable employees to improve their spiritual needs in the workplace, and form a unique organizational spirit and culture, which is the driving force for the sustainable development of organizations. It can be seen that the spiritual leadership has a diffusion role, which can expand from the personal inner life and spiritual care of the leaders to the individual staff and the whole organization playing the role of a “beacon” (Yang et al., 2014). At the same time, interpersonal trust has a significant mediating effect on spiritual leadership and employee morale, which shows that recognition and trust play a vital role in working relationships on one hand; and on the other hand, improving employee morale should rely more on the improvement of working atmosphere and environment. The application of spiritual leadership is beneficial to atmosphere. Based on the improvement of organizational cohesion, employees’ working attitude and motivation will be more effective. Their intrinsic motivation is stimulated, which becomes more passionate.

By studying the direct effect of spiritual leadership on employee morale and the indirect effect of self-efficacy and interpersonal trust chain intermediary, this paper analyzes the mechanism and mode of spiritual leadership to improve employee morale. It discusses the application of spiritual leadership to organizational culture and strategy development. It enriches the content of workplace spirituality research, and provides evidence support and conclusion suggestions for the improvement of employee morale and the construction of organizational atmosphere in the context of Chinese cultural situation.

### 4.2. Recommendations

Spiritual leadership helps to improve employee morale from two aspects: intrinsic motivation and the trust working atmosphere. Improving the workplace spiritual level will develop a unique competitive advantage for the organizations, conducive to dealing with the complex and changeable business environment and forming the driving force of sustainable development. For the sustainable development of organizations, the following suggestions are put forward:

1. Pay attention to spiritual factors in organizations to meet the spiritual needs of the employees. The spiritual content in the organization requires the participation of all members. And the formation of vision planning, hope culture, and altruistic love values require leaders’ involvement in the organization. To improve the spiritual leadership of the organization, we should emphasize the important role of spiritual factors such as self-reflection and mindfulness in the development of leadership to guide the organization to implement the practical activities of spiritual leadership. In Sounds True (A multimedia publishing company in Colorado, United States), employees are encouraged to “work with their complete selves and work with others”; at 11 a.m., the clock rings for 15 min for meditation or silence (Fry et al., 2017). Some organizations support conversations between employees about soul needs, personal achievements and spiritual aspirations.
2. Carry out practical activities to improve the internal spirit in the workplace. It is suggested that in organizing various activities, attention should be paid to the construction of members’ personal spiritual life and trust atmosphere in the organization. Practical activities for internal life, such as reflection on values, can not only improve the spiritual needs of employees and stimulate their intrinsic motivation in the process of work; moreover, it has developed a unique spirit and energetic atmosphere in the organization, enhanced the sense of belonging of employees, and is conducive to improve the quality of organizational services.
3. In this study, spiritual leadership plays a positive role, and most scholars reached positive conclusions. In the process of leaders in organization ship building, we should pay full attention to the inner life and good quality of leaders and make an objective evaluation of their belief enthusiasm. The spiritual needs and helpful qualities of the candidates should be important dimensions in choosing leaders in organization. While many organizations choose leaders with better performance in reality.

The theory and practice of spiritual leadership in China are both in the initial stage of development. With the enrichment of material life and the increasing spiritual needs of organizational employees, spiritual leadership will become an important part of organizational culture and be well valued and promoted. At the same time, spiritual leadership can be combined with organizational behavior to conduct new discussions, such as the impact of the CEO’s social responsibility on organizational performance and employee performance (Khan et al., 2022; Liu et al., 2022).

## 5. Limitations and future research

1. Due to the limitation of objective conditions, the questionnaire survey is randomly distributed to alumni *via* the questionnaire star website. There is no excessive condition setting for the respondents. The paired questionnaire content should be designed for the research objects of specific industries or characteristics, and the sample size should be expanded in future study. In that case, it is believed that more rich conclusions will be drawn.
2. The analysis of the influence mechanism of spiritual leadership can focus on the research of organizational work atmosphere. The combination of in-depth interviews and questionnaires can be organized for specific cases, which can help excavate and discover the unique spiritual content of the organization. Targeted analysis can also be conducted for leaders in different industries and at different levels. Since it involves subjective cognition, the application situation of some questionnaire items may be different, and it is also very important to develop the content of the questionnaire under the Chinese culture situation. The development of such a questionnaire also needs to be done through in-depth interviews.
3. In the research model, only gender factors in the control variables negatively impact self-efficacy and employee morale; women’s self-efficacy and employee morale are lower than men. This may be related to the cultural situational factors. In the later research process, we can study the demographic variables of spiritual leadership through interviews and theoretical analysis.

## Data availability statement

The raw data supporting the conclusions of this article will be made available by the authors, without undue reservation.

## Ethics statement

The studies involving human participants were reviewed and approved by Ludong University Research Ethics Review Committee. The patients/participants provided their written informed consent to participate in this study.

## Author contributions

All authors listed have made a substantial, direct, and intellectual contribution to the work and approved it for publication.

## Funding

This research was supported by the MOE (Ministry of Education in China) Youth Foundation Project of Humanities and Social Sciences (Project No. 19YJC630180); the Youth Foundation of Shandong Natural Science Foundation (Project No. ZR2022QG052); the Open Fund Project of Zhejiang Modern Service Research Center (Project No. SXFJZ202202).

## Conflict of interest

The authors declare that the research was conducted in the absence of any commercial or financial relationships that could be construed as a potential conflict of interest.

## Publisher’s note

All claims expressed in this article are solely those of the authors and do not necessarily represent those of their affiliated organizations, or those of the publisher, the editors and the reviewers. Any product that may be evaluated in this article, or claim that may be made by its manufacturer, is not guaranteed or endorsed by the publisher.

## Footnotes

^1^Neijuanis a net word in China, it comes from "involution," which means internal competition in an organization.

^2^996 is a net word in China. It means working from 9 a.m. to 9 p.m. for 6 days a week.

^3^LinkedIn (2022). LinkedIn Global Talent Trend Report, 2022 https://max.book118.com/html/2022/0722/5021341001004312.shtm, 37(1):86–96.

^4^ https://www.wjx.cn/
